# Roles of structural plasticity in chaperone HdeA activity are revealed by ^19^F NMR†
†Electronic supplementary information (ESI) available: 1D ^19^F NMR and 2D ^1^H–^15^N HSQC spectra. See DOI: 10.1039/c5sc04297f


**DOI:** 10.1039/c5sc04297f

**Published:** 2015-12-03

**Authors:** Zining Zhai, Qiong Wu, Wenwen Zheng, Maili Liu, Gary J. Pielak, Conggang Li

**Affiliations:** a Key Laboratory of Magnetic Resonance in Biological Systems , State Key Laboratory of Magnetic Resonance and Atomic and Molecular Physics , National Center for Magnetic Resonance in Wuhan , Wuhan Institute of Physics and Mathematics , Chinese Academy of Sciences , Wuhan , P. R. China . Email: conggangli@wipm.ac.cn; b University of Chinese Academy of Sciences , Beijing , P. R. China; c Department of Chemistry and Department of Biochemistry and Biophysics , University of North Carolina , Chapel Hill , NC , USA; d Lineberger Comprehensive Cancer Center , University of North Carolina , Chapel Hill , NC , USA

## Abstract

HdeA, a minimal ATP-independent acid chaperone, is crucial for the survival of enteric pathogens as they transit the acidic (pH 1–3) environment of the stomach. Although protein disorder (unfolding) and structural plasticity have been elegantly linked to HdeA function, the details of the linkage are lacking. Here, we apply ^19^F NMR to reveal the structural transition associated with activation. We find that unfolding is necessary but not sufficient for activation. Multiple conformations are present in the functional state at low pH, but the partially folded conformation is essential for HdeA chaperone activity, and HdeA's intrinsic disulfide bond is required to maintain the partially folded conformation. The results show that both disorder and order are key to function. The ability of ^19^F NMR to reveal and quantify multiple conformational states makes it a powerful tool for studying other chaperones.

## Abstract 2

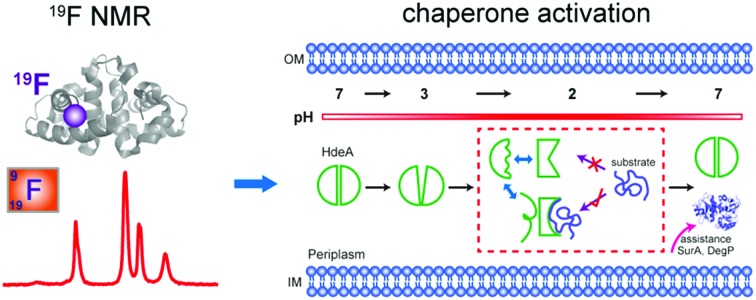
Multiple conformations of acid chaperone HdeA and their roles in activity.

## Introduction

Conditionally disordered proteins, which may comprise the majority of intrinsically disordered proteins, convert between ordered and disordered conformations.^1^ The acid-activated chaperone HdeA, from the periplasm of pathogenic bacteria, is a typical example.^2^ Transit of these bacteria through the acidic mammalian stomach (pH 1–3) is a huge challenge.^3^ HdeA, one of several protective systems that gut-resident *Escherichia coli* strains have evolved to counter this stress,^2^ prevents the aggregation of periplasmic proteins brought about by the drop in pH.^4^,^5^

At neutral pH, HdeA is an inactive, structured dimer.^5^,^6^ Once in the stomach HdeA dissociates into an active partially unfolded monomer, binds denatured substrates, and prevents their aggregation.^4^,^7^ Upon entering the pH-neutral small intestine, HdeA releases its substrates and returns to a folded dimer.^8^ The released proteins are refolded with the assistance of chaperones SurA and DegP, which are also protected by HdeA.^9^

As a small, ATP-independent acid chaperone, HdeA is an attractive model for studying the relationships between disorder, client specificity and chaperone activity.^1^ Recently, Foit *et al.* constructed a partially unfolded, active variant at neutral pH by replacing two aspartic acid residues, suggesting that activation requires unfolding.^10^ Nevertheless, details about activation are lacking.^11^,^12^ Do some parts remain structured while others are unstructured? What is the role of the intramolecular disulfide bond? Additionally, the mode of substrate binding and the dynamics of active HdeA are entirely unknown.

^19^F NMR is a powerful tool for studying protein dynamics and interactions involving complex systems like proteins both *in vitro* and in cells.^13^,^14^ It is also widely used for ligand selection in pharmacology.^14^,^15^ In addition to its high sensitivity, spectral simplicity and low background, ^19^F labelling is sensitive to the surroundings, because the nucleus exhibits a large chemical shift range. Hence ^19^F NMR can be an effective probe for monitoring protein conformational changes and ligand binding.

We use ^19^F labelling to quantify the local unfolding of HdeA during acid activation. Combining experiments on structural stability and activity, we show that unfolding is necessary but insufficient for chaperone activation. We also show that multiple dynamic conformations of HdeA are present at low pH, that a partially folded conformation is required for activity and that the intramolecular disulfide-bond is required to maintain the partially folded active form. In addition, the disulfide plays a critical role in HdeA refolding. Our proposed mechanism reveals the link between structural plasticity and function for conditionally disordered proteins, especially molecular chaperones.

## Results

### ^19^F NMR provides quantitative insight into unfolding and activation

**Fig. 1:**
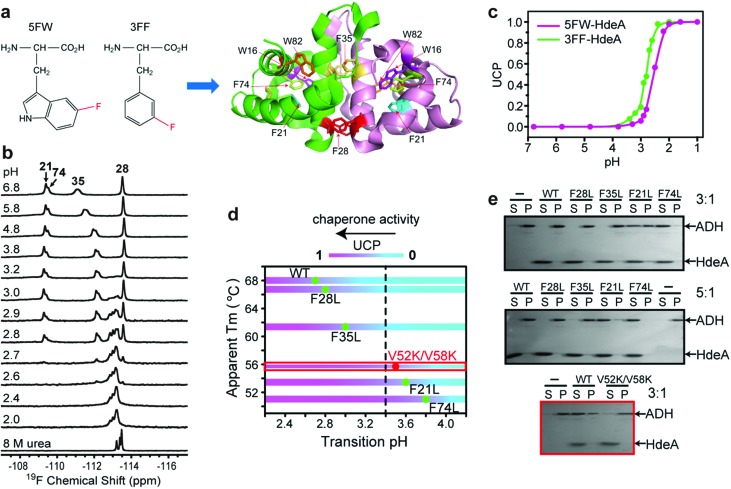
Relationship between pH-induced conformation transition and function. (a) ^19^F-labelled tryptophan (5FW) and phenylalanine (3FF) and the labelling sites on the dimer (PDB: ; 1DJ8). (b) ^19^F spectra of 3FF-labelled HdeA at several pH values and in 8 M urea. (c) Unfolded conformation population (UCP) of 5FW- and 3FF-labelled HdeA as function of pH (spectra of 5FW-labelled HdeA at different pH values are shown in Fig. S4†). (d) Correlation between transition pH and apparent melting temperature, *T*_m_. As the pH decreases, the population of the unfolded conformation increases from 0 (cyan) to 1 (magenta), accompanied by growth in chaperone activity, except for V52K;V58K (red box). (e) Chaperone activity assays on WT HdeA and variants at pH 3.4 at HdeA : substrate ADH mole ratios of 3 : 1 and 5 : 1, and for HdeA WT and the V52K;V58K at pH 2 at a 3 : 1 ratio.

The acid unfolding of HdeA is key to its activation.^10^ To investigate the mechanism, we first studied the ^1^H–^15^N HSQC spectrum of uniformly ^15^N-enriched HdeA, but it is difficult to assign the overlapped and broad cross peaks at low pH (Fig. S1†); we then turned to simpler 1D ^19^F NMR.^13^ We labelled the two tryptophan residues with 5-fluorotryptophan (5FW) and its four phenylalanine residues with 3-fluorophenylalanine (3FF) (Fig. 1a). W16 and F21 are situated (Fig. 1a) in α-helix H1 near the N-terminus, F74 and W82 in α-helix H4 are near the C-terminus, and all these residues are buried in the hydrophobic core.^5^ F28 and F35 comprise part of the hydrophobic dimer interface, which binds denatured substrates at low pH.^7^ The observation of ^19^F resonances with characteristic chemical shifts indicates correct labelling. Resonance assignments were achieved with variant proteins (Fig. S2†). The good overlay of HSQC spectra (Fig. S3†) suggests that the labelling is non-perturbing.

To reveal the conformational transition from folded dimer at neutral pH to unfolded monomer at low pH, a series of ^19^F spectra were acquired at 37 °C between pH 6.8 and pH 1.0 (Fig. 1b and S4†). For both 5FW- and 3FF-labelled HdeA, a new resonance appeared near pH 3. The intensity of the original resonance gradually decreased, disappearing completely at pH 2. Comparisons to spectra of the 5FW- and 3FF-labelled HdeA in 8 M urea, indicate that the new resonance arises from an unfolded conformation. The population of the unfolded conformation (UCP) was obtained at different pH values (Fig. 1c) by integrating the original resonance (folded conformation) and the new resonance (unfolded conformation). The transition pH can be read from the curve.

Additionally, ^19^F resonances differentially respond to pH, revealing distinct local conformational changes. The resonance shifts of W82 and F35 show the sensitivity of these sites to the decrease in pH from 6.8 to 3.0, before new resonances from the unfolded form appear (Fig. 1b). Notably, the resonance of F35 situated at the dimer interface changes in a stepwise manner to the disordered chemical shift (8 M urea), while the resonance from F28, located at the bottom of interface, is unchanged down to pH 2.8. The shift change of F35 above pH 3.0 suggests a change in the chemical environment around this residue, possibly due to protonation of nearby residue, E37, with p*K*_a_ > 5 or dimer dissociation occurring near F35 involving the charged loop between α-helices H2 and H3.^16^ This result supports the observation that aspartic- and glutamic-acid charge neutralization destabilizes the dimer prior to unfolding.^17^

After constructing single variants to facilitate 3FF resonance assignment, we recorded ^19^F spectra as a function of pH (Fig. S5†). Interestingly, the four variants show higher pH midpoints for unfolding compared to WT (Fig. 1d). The midpoints of the F21L and F74L variants increased to pH 3.6 and 3.8, respectively, approximately one unit higher than WT. These results imply that leucine substitutions destabilize the folded structure. This destabilization is possibly due to disruption of the aromatic cluster involving F21, F74 and W15.^18^ To assess the destabilization, we used circular dichroism spectropolarimetry to measure the midpoints of thermal denaturation (Fig. S6† and 1d). As expected, the higher the transition pH, the lower the melting temperature (Fig. 1d), meaning that thermal stability and pH stability are linked.

To determine if the destabilized variants have chaperone activity at a higher pH compared to WT, we performed activity assays at pH 3.4, where the F74L variant is almost completely unfolded, the F21L variant is about half unfolded, and F35L, F28L variants and the wild-type protein remain mostly folded (Fig. 1d). The data (Fig. 1e) demonstrate that both the F74L and F21L variants suppress the aggregation of alcohol dehydrogenase (ADH), a typical substrate, at a mole ratio of 5 : 1. ADH aggregation was partially suppressed by the F21L variant at a ratio of 3 : 1. However, no matter what the ratio, ADH precipitated almost completely in the presence of the F35L and F28L variants and the wild-type protein. This result proves that unfolding is necessary for activation.

Paradoxically, the V52K;V58K double variant has greatly reduced chaperone-like activity, but its acid-induced structure transformation is only slightly affected.^17^ We also demonstrated (Fig. S7†) that the acid-induced transformation of the variant is similar to that of the wild-type protein. However, the wild type protein is partially unfolded at acidic pH (Fig. S1†), but the variant is completely unfolded. Despite possessing a transition pH similar to that of the F21L variant, the V52K;V58K variant lacked activity even when completely unfolded (Fig. 1e). We conclude that unfolding is necessary but insufficient for chaperone activation.

### Multiple dynamic conformations at low pH

**Fig. 2:**
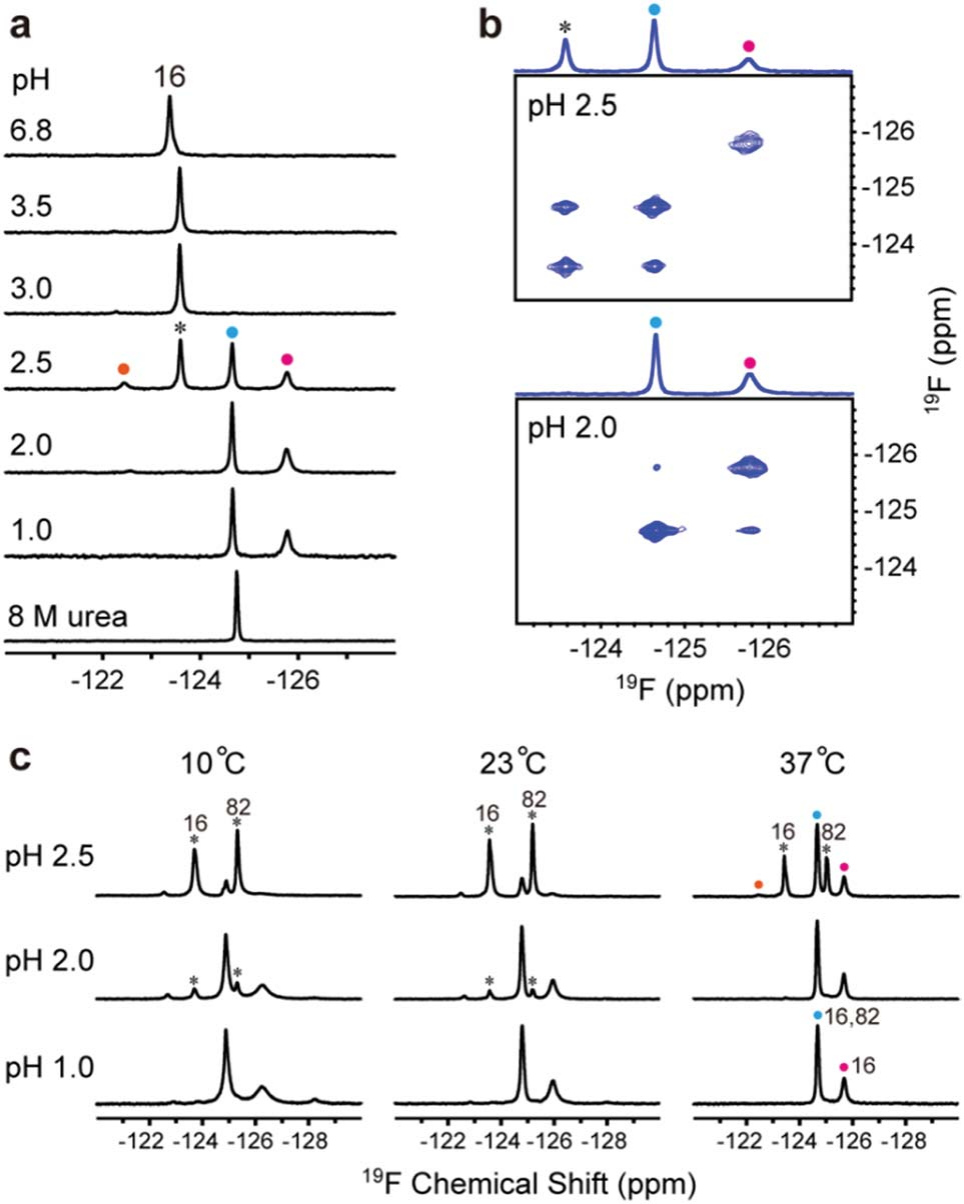
Identifying multiple distinct conformations. (a) ^19^F spectra of 5FW-labelled W82F variant at different pH values and in 8 M urea. The black asterisk indicates the original resonance at –123.6 ppm (W16). Coloured circles indicate new resonances from W16 at –122.5 ppm (orange), –124.6 ppm (blue) and –125.8 ppm (magenta). (b) Regions of the ^19^F EXSY spectrum (500 ms mixing time) of the 5FW labelled W82F variant at pH 2.5 and pH 2.0. One-dimensional projections are shown above the spectra. (c) Spectra of 5FW-labelled WT HdeA at three temperatures and pH values. The black asterisks indicate the original resonances at –123.6 ppm (W16) and –125.2 ppm (W82). A new peak (blue circles) comes from both W16 and W82 resonances. The other new resonances are from W16.

To understand the relationship between conformation and activity, we acquired spectra of the tryptophan-labelled W82F and W16F variants at pH 2.5. At 37 °C, the W82F variant exhibits four peaks, including one from the folded form, suggesting that W16 gives rise to three new resonances at this pH (Fig. 2a). In contrast, W82 in the W16F variant gives rise to a new single resonance at –124.6 ppm that overlaps resonances from W16 (Fig. S8†). However, the urea denaturation is different from acid unfolding; fewer ^19^F resonances are observed and only a single sharp resonance is observed in 8 M urea (Fig. S9†). These data suggest that HdeA possesses residual structure and is conformationally heterogeneous at pH 2.5.

To assess the dynamics of the various conformations, we acquired ^19^F EXSY^19^ spectra at 37 °C (500 ms mixing time) at pH 2.5 and 2.0. A cross peak between the original resonance and the new resonance at –124.6 ppm appeared at pH 2.5, as does a very weak cross peak between the new resonances at –124.6 ppm and –125.7 ppm at pH 2.0 (Fig. 2b). These data suggest that multiple conformations of HdeA present at low pH are in dynamic exchange on a 100 ms, or longer, timescale.

In addition, we collected spectra of the 5FW-labelled wild-type protein at low pH values and three temperatures (Fig. 2c). The numbers and intensity of the ^19^F resonances vary with temperature, suggesting that the conformational heterogeneity of active HdeA is affected by both temperature and pH. A fourth new conformation is observed at 10 °C and pH 1.0. No structured dimer remains at pH 1.0 at any temperature, yet two conformations can be detected. These conformations give rise to the narrow and broad resonances.

### Partially folded conformer is essential for activity

**Fig. 3:**
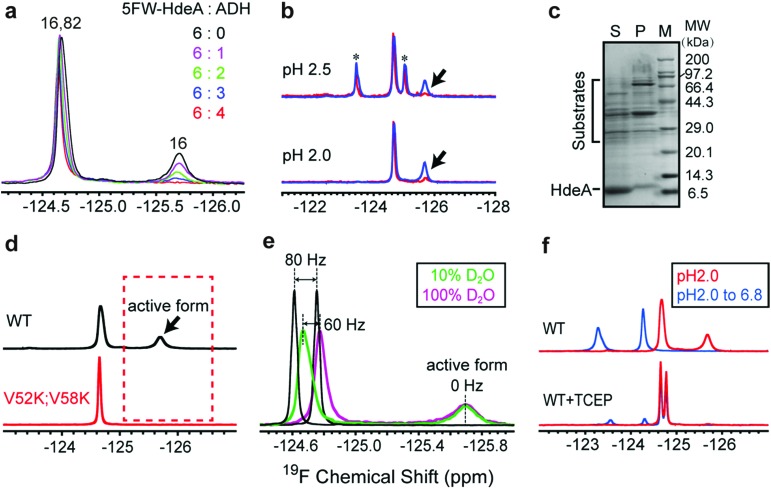
Partially folded form is essential for activity. (a) ^19^F spectra of 5FW-labelled WT protein upon adding substrate ADH (mole ratio 5FW-HdeA : ADH from 6 : 0 to 6 : 4) at pH 2.0. The downfield peak is from both W16 in the unfolded form and W82 in the unfolded part of the protein. The upfield resonance is from W16 in the partially folded form. (b) Overlaid spectra from purified HdeA (blue) and an HdeA-containing supernatant from *E. coli* (DE3) periplasmic extracts (red) at pH 2.5 and 2.0. Arrows indicate the binding competent conformation. The black asterisks indicate the original resonances. (c) SDS-PAGE analysis of supernatant (S) and pellet (P) from periplasmic extracts at pH 2.0. Molecular weight standards (M) are also shown. HdeA and substrates were identified in the supernatant. (d) Comparison of spectra of WT and V52K;V58K at pH 2.0. The arrow indicates the active form inferred from the absence of chaperone activity for the V52K;V58K variant. (e) Spectra of 5FW-labelled WT and free 5FW (black) in 10% (v/v) D_2_O and 100% D_2_O at pH 2.0. Frequency changes induced upon transfer are shown. (f) Spectra of WT and WT reduced with tris(2-carboxyethyl)phosphine·HCl [(TCEP) at pH 2.0] after return to pH 6.8. Identical HdeA concentrations were used in each panel, and spectra were processed identically.

To assess the functional roles of the conformations present at low pH, we added the substrate ADH to the wild-type protein at pH 2.0 (Fig. 3a). The area under the broader resonance at –125.8 ppm decreases, but that of the narrower one at –124.6 ppm is only slightly perturbed. This observation suggests that the conformation represented by the broader resonance binds the substrate. That is, the resonance is broad and difficult to detect because the substrate-bound form has a large effective molecular weight, or the bound and free forms exchange at a rate approximately equal to the difference in their resonance frequencies. To obtain more information, we studied proteins smaller than ADH (35 kDa) at pH 2: the F30H variant of the B1 domain of protein G (GB1, 6 kDa), α-synuclein (15 kDa) and calmodulin (CaM, 16 kDa). These proteins are unfolded at pH 2.0. The ^1^H–^15^N HSQC spectra of GB1, α-synuclein and CaM indicate that HdeA binds CaM, but not GB1 and α-synuclein at pH 2. The ^19^F and ^1^H–^15^N HSQC spectra of HdeA in the presence of these model proteins support the same conclusion (Fig. S10–S15†). These observations suggest that HdeA has some substrate specificity. Although CaM is smaller than ADH, the broad ^19^F resonance still disappears, as do some cross-peaks in the HSQC spectra in CaM titrations, suggesting that the disappearance is mainly due to the presence of an intermediate chemical exchange rate between bound and free HdeA. Other native substrates in the *E. coli* (DE3) periplasmic extract were also tested (Fig. 3b). Client periplasmic proteins, including RbsB^4^ (29 kDa), were identified by SDS-PAGE analysis (Fig. 3c).

To determine why the V52K;V58K variant is inactive despite being unfolded, we labelled it with 5FW and acquired spectra at pH 2.0. The absence of the broader resonance compared to WT (Fig. 3d) is consistent with our conclusion that the conformation represented by the broad resonance is crucial for chaperone function. The single narrow peak represents a globally disordered conformation, as inferred from analysis of ^19^F and ^1^H–^15^N HSQC spectra (Fig. S7†).

To reveal some of the structural features of the active state we assessed the solvent exposure of the labelled side chains by measuring the effect of changing the solvent from H_2_O to D_2_O on the ^19^F chemical shifts.^20^–^22^ Free 5FW is completely solvent exposed, and its chemical shift changes by 80 Hz upon transfer (Fig. 3e). For active WT, the narrower resonance at –124.6 ppm shifts ∼60 Hz, while the broader resonance at –125.8 ppm does not shift, suggesting that the tryptophan residue in the conformation represented by the narrow resonance has a large exposure to solvent, but the resonance from the active conformation is buried. The observation of a few cross peaks in ^1^H–^15^N HSQC spectra of HdeA in 100% D_2_O at pH 2.0 also suggests that parts of unfolded HdeA remain structured, protecting those amide protons from solvent (Fig. S16†). We conclude that, regardless of the presence of substrates, the region around W16 in the active conformation is partially folded, and that the disordered conformation (reflected by the downfield peak from W82 and W16) exists simultaneously.

Given that W16 lies near the intramolecular disulfide between C18 and C66, we hypothesized that there is a relationship between the partially folded active structure and the disulfide.

As expected, reducing the disulfide greatly reduces chaperone activity (Fig. S17†), the protein loses the spectral signature of the active form at pH 2.0 and cannot refold to an ordered dimer at pH 6.8 (Fig. 3f).

## Discussion

The human stomach maintains a pH of 1 to 2 as a natural barrier against infection by food-borne pathogens. The pH increases to between 2 and 4 after eating.^23^ Enteric bacteria employ two homologous acid-chaperones, HdeA and HdeB, to protect acid-denatured periplasmic proteins. The function of HdeB is optimal at pH 4 as a dynamic folded dimer,^24^ while the function of HdeA is optimal at pH 2 as a partially unfolded monomer. Our data show that the folded to unfolded transition occurs between pH 3 and 2. Our activity data, and those of others,^10^ demonstrate that unfolding is necessary for chaperone activation. We have gone further by using NMR to quantify the population of the unfolded conformation, showing that the extent of unfolding determines the substrate-protecting ability.

Tapley and Dickson *et al.* proposed that HdeA populates conformational ensembles that depend on the structure of its substrates.^7^,^25^ A genetically encoded photocrosslinker was used to capture *in vivo* client periplasmic proteins of HdeA.^9^ In general, the ability to bind different substrates potentially requires structural adaptations.^26^,^27^ Indeed, our observation of multiple dynamic conformations (Fig. 2), explains this adaptability. The conformational heterogeneity is intrinsic to active HdeA, enabling the chaperone to respond to a variety of substrates.

Two dominant conformations were detected by ^19^F NMR at physiological temperature. However, the chemical shift and intensity of the unfolded form remains constant in the presence of substrate, suggesting that the partially folded form binds substrate. Nevertheless, our data cannot completely rule out binding by the unfolded species. Replacing two hydrophobic residues with positively charged lysines greatly reduced chaperone activity despite having little influence on the folded dimer to unfolded monomer transition. Although a previous study concluded that the reduced exposure of hydrophobic surfaces prevented substrate binding,^28^ the in-depth reason as revealed here is that the mutations convert multiple conformations into a globally disordered structure. The absence of the partially folded form demonstrates that unfolding alone does not bring about activity. Instead, the residual folded structure is required to promote HdeA chaperone activity.

**Fig. 4:**
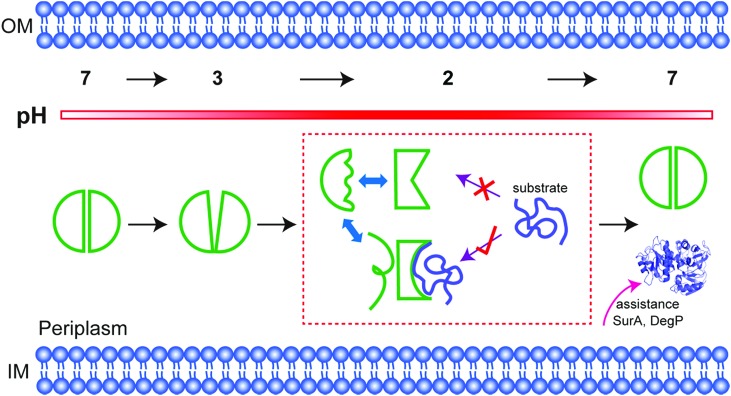
Activation mechanism. On decreasing the pH from 7 to 3, HdeA becomes a loosened dimer, and dissociation begins at the top of the interface. At pH 2, the protein becomes an unfolded monomer with several distinct conformations that exchange on the chemical shift timescale. Only the partially folded conformation reflected in the region of the disulfide bond can bind and then release the denatured substrate, which is refolded with assistance from other chaperones at neutral pH. The more disordered of the two dominant conformations may protect the active form.

In fact, among the six ^19^F labelling sites in representative structural regions, only W16 shows conformational heterogeneity and a partially folded form at low pH; the others all sense the disordered regions. Consistent with this idea, the partially folded structure is directly related to the intramolecular C18–C66 disulfide bond near W16. Once the bond is broken, HdeA loses activity and cannot refold to a structured dimer. Therefore, the disulfide regulates the local conformational heterogeneity that is essential to substrate binding and provides the structural basis for refolding. Thus, the disulfide is the structural key for HdeA to act as an acid chaperone. Importantly, a disordered form sensed by W16 is also always present, the necessary existence of which is suspected to protect or support the active form (Fig. 4). This new structural mechanism, order in disorder, improves our understanding of how these disparate structures both play a role in molecular chaperones.

## Conclusions

We provide structural information for HdeA in its functional state at atomic resolution. The distinctive quantitative insight into the population of both partially folded and unfolded conformations of HdeA provided by ^19^F NMR allowed us to define the mechanism of this chaperone. The ability to detect multiple conformations and their exchange dynamics shows that ^19^F NMR is a powerful probe of the activation mechanisms of conditionally disordered chaperones under stress.

## Experimental section

### Materials and methods

#### Protein labelling and purification

The pET21a plasmids containing the genes for HdeA and its variants were transformed into BL21 (DE3) *E. coli* cells. Cells harbouring the plasmid were selected with 100 μg ml^–1^ ampicillin. Two 1 L cultures were grown at 37 °C in minimal media containing 1 g of ^15^NH_4_Cl at 37 °C. For 3FF labelling, 70 mg d,l-*m*-fluorophenylalanine, 60 mg l-tyrosine, 60 mg l-tryptophan and 0.5 g glyphosate were added when the absorbance at 600 nm (OD600) reached ∼0.4.^13^ For 5FW labelling, 60 mg 5-fluoroindole was added when the OD600 reached ∼0.9.^29^ Both cultures were shaken at 37 °C until the OD600 reached ∼1.0, at which time the inducer isopropyl β-d--​1-​thiogalactopyranoside was added to a final concentration of 1 mM. Cells were grown for an additional 16 h at 20 °C and harvested by centrifugation.1--​1-​thiogalactopyranoside was added to a final concentration of 1 mM. Cells were grown for an additional 16 h at 20 °C and harvested by centrifugation.thiogalactopyranoside was added to a final concentration of 1 mM. Cells were grown for an additional 16 h at 20 °C and harvested by centrifugation.

The cell pellet was resuspended in Q Sepharose buffer A (20 mM Tris, pH 8.0, 0.5 mM ethylenediaminetetraacetic acid) for sonication. The supernatant was collected after centrifugation. Purified ^15^N-enriched GB1, α-synuclein and calmodulin, ^19^Calmodulin were obtained as described.^30^,^31^

Periplasmic extracts from *E. coli* containing 5FW-labelled HdeA were prepared by resuspending the cell pellets in citrate buffer (10 mM citric acid, 50 mM NaCl, pH 6.8) and 1 mg ml^–1^ polymyxin sulphate,^32^ swirling for 1 h at 4 °C, followed by centrifugation to remove the cytoplasmic fraction and cell debris.

#### Circular dichroism

Data were acquired on a Chirascan spectropolarimeter using quartz cuvettes with a path length of 0.1 cm. Spectra were recorded using 20 μM HdeA (WT, F74L, F21L, F28L, F35L and V52K;V58K) in 10 mM potassium phosphate buffer, pH 6.8. The proteins were labelled with 3FF except for the V52K;V58K variant, which was labelled with 5FW. For melting curves, the CD signal at 222 nm was monitored at a heating rate of 0.3 °C min^–1^. Labelling has a negligible effect on the stability.

#### pH titration

The protein sample (0.2 mM to ∼1.0 mM) in the titration buffer (10 mM citric acid, 50 mM NaCl, 0% D_2_O, 10% D_2_O or 100% D_2_O) was adjusted to the desired pH value from 6.8 to 1.0 by stepwise additions of HCl or NaOH (prepared in 100% D_2_O for D_2_O assays). The pH meter was standardized with pH 2.00, pH 4.01 and pH 7.00 commercial standards. The protein solution pH was nearly unaffected at 10 °C and from 25 °C to 37 °C.

#### HdeA activity

Chaperone-like activity was assessed by following the appearance of substrate protein in the supernatant instead of the pellet.^4^ Aggregation assays were performed by diluting ADH (Sigma) to a final concentration of 10 μM into the aggregation buffer [10 mM citric acid, 50 mM NaCl, 150 mM (NH_4_)_2_SO_4_] with or without 30 μM or 50 μM of HdeA (WT, F28L, F35L, F21L and F74L) at pH 3.4. The same conditions were used for assessing WT and V52K;V58K at pH 2.0. The presence of substrate protein in the supernatants or pellets was assessed by SDS-PAGE after incubation at 37 °C for 1 h followed by centrifugation at 14 000*g* for 10 min. Pellets were resuspended in H_2_O to a volume equal to that of the supernatant prior to loading the gel.

#### ^19^F NMR and substrate-binding experiments

Spectra were acquired on a Bruker 600 MHz spectrometer equipped with a 5 mm H/F/(C, N) triple resonance cryoprobe at 37 °C unless stated otherwise. A sweep width of 11 kHz was used to acquire up to 2048 transients with a 2 s duty cycle delay for 1D spectra. For EXSY spectra, the width was 3 kHz with a 0.45 s acquisition time and a 3 s relaxation delay. Chemical shifts were referenced to trifluorotoluene at –63.72 ppm. Spectra were identically processed with Topspin 3.2 software. Binding assays were performed at pH 2.0 by adding an increasing amount of ADH (up to 0.2 mM) to 0.3 mM HdeA (WT and W82F) in buffer (10 mM citric acid, 50 mM NaCl, 10% D_2_O). The sample remains clear until the molar ratio HdeA : ADH increases to 3 : 2, when HdeA is saturated. The conditions for the ^1^H–^15^N HSQC spectra were identical to those used for the ^19^F NMR experiments, unless indicated otherwise. The high HdeA concentration used for NMR has the same acid-induced structure transition as the low concentration used in the biochemical assays. Protein concentration independence has been reported.^30^

## Supplementary Material

Supplementary information
