# Supplementary material for: Roles of structural plasticity in chaperone HdeA activity are revealed by 19F NMR
Source: Chem Sci. 2015 Dec 3;7(3):2222–8. doi: 10.1039/c5sc04297f (PMC5975942; doi:10.1039/c5sc04297f)
Supplement: Supplementary file 1 [file SC-007-C5SC04297F-s001.pdf]

Electronic Supplementary Material (ESI) for Chemical Science.
This journal is © The Royal Society of Chemistry 2015

Supplementary Information for:

Roles of structural plasticity in chaperone HdeA activity are revealed by ^{19}F NMR

Zining Zhai[a,b], Qiong Wu[a], Wenwen Zheng[a,b], Maili Liu[a], Gary J. Pielak[c,d], and Conggang Li[a,*]

[a]Key Laboratory of Magnetic Resonance in Biological Systems, State Key Laboratory of Magnetic Resonance and Atomic and Molecular Physics, National Center for Magnetic Resonance in Wuhan, Wuhan Institute of Physics and Mathematics, Chinese Academy of Sciences, Wuhan, P. R. China. [b]University of Chinese Academy of Sciences, Beijing, P. R. China. [c]Department of Chemistry and Department of Biochemistry and Biophysics, University of North Carolina, Chapel Hill, NC, U.S.A. and [d]Lineberger Comprehensive Cancer Center, University of North Carolina, Chapel Hill, NC, U.S.A.

[*]Corresponding author: congganglі@wipm.ac.cn

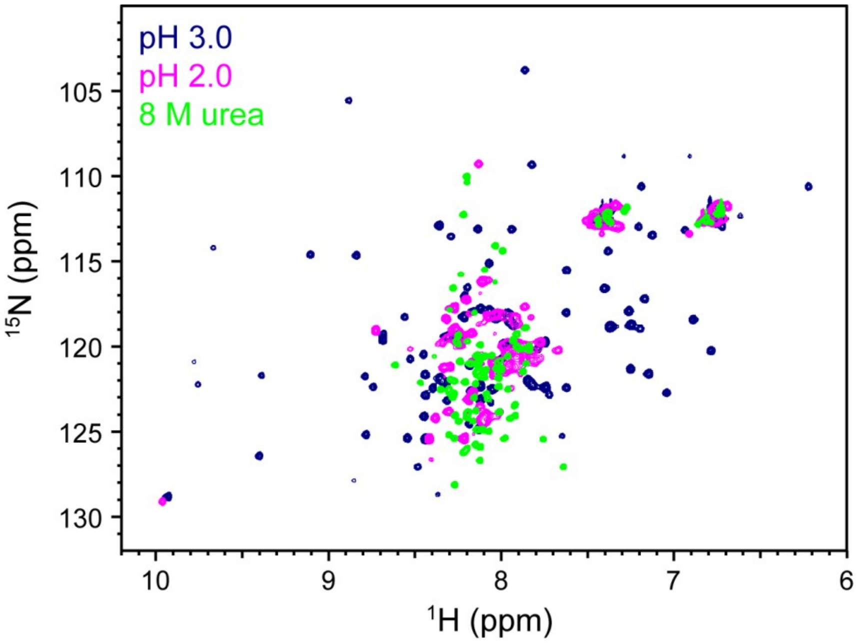

Figure S1. ^{1}H-^{15}N HSQC spectra of HdeA at pH 3.0 (blue), pH 2.0 (magenta) and in 8 M urea (green). Dissociation to the unfolded monomer at pH 2.0 causes severe overlap and broadening compared to the structured dimer. Acid unfolded HdeA possesses residual structure, which is in contrast to the complete disorder in 8 M urea.

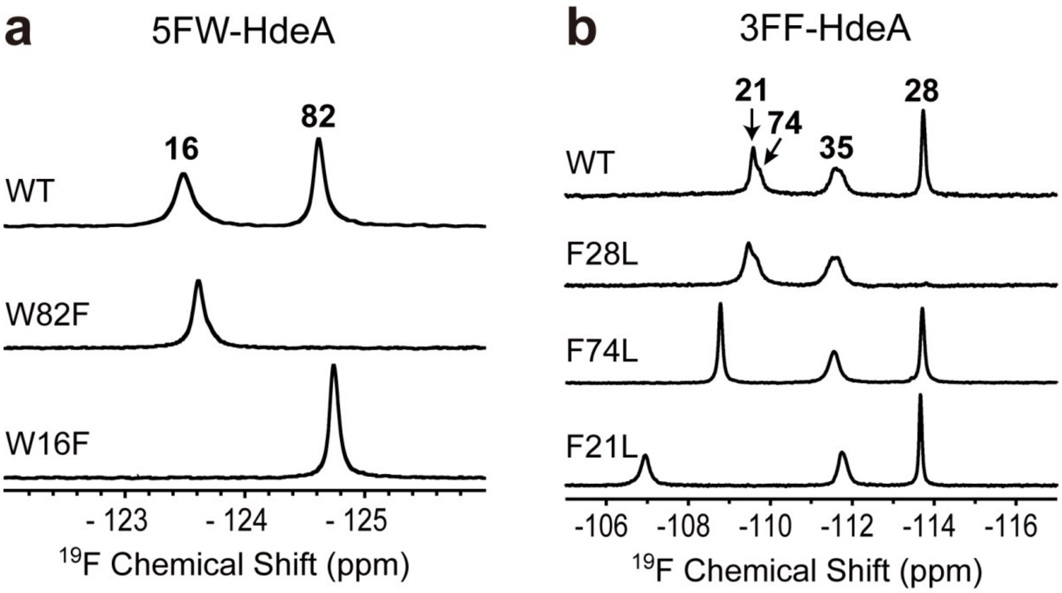

Figure S2. ^{19}F NMR spectra and assignments of 5-fluoro-tryptophan (5FW) (**a**) and 3-fluoro-phenylalanine (3FF) (**b**) labelled HdeA at neutral pH. Resonances of F21 and F74 overlap in the 3FF-WT spectrum. Resonances from F21L and F74L variants differ in chemical shift from those of the wild-type protein (**b**), suggesting local structural perturbation by mutation. Inspection of the crystal structure shows that phenylalanines 21 and 74 are stacked. Therefore, we expect larger shift changes for the F-to-L variants, as is observed. We based the individual assignments on the width of the resonances. Replacing F74 with a leucine leaves a sharp resonance, which we assign to F21. Replacing F21 with a leucine leaves a broad resonance, which we assign to F74.

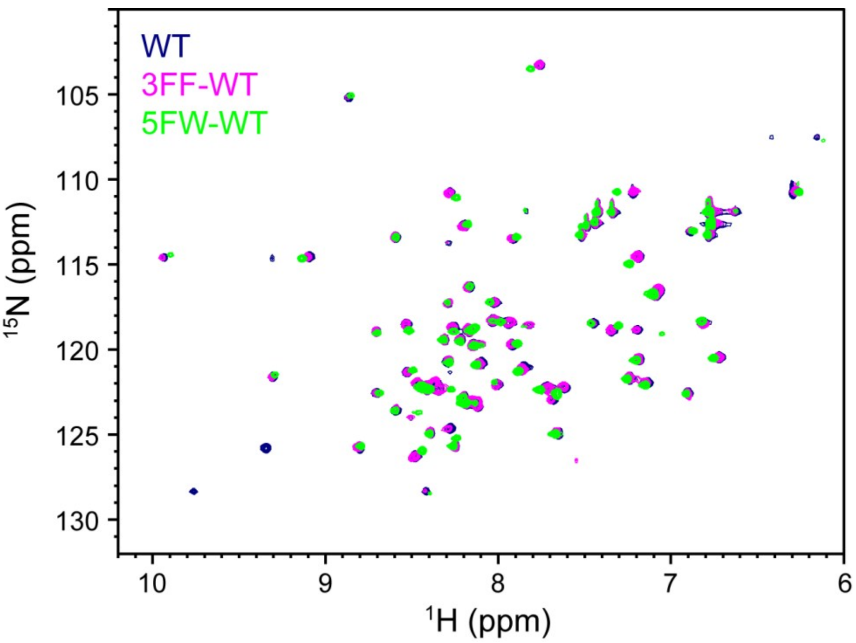

Figure S3. ^{1}H-^{15}N HSQC spectra (pH 6.8) of ^{15}N-enriched WT HdeA; 3FF-labelled, ^{15}N-enriched WT HdeA, and 5FW-labelled, ^{15}N-enriched WT HdeA. The overlap of crosspeaks from the different forms of the protein suggests that labelling does not perturb the structure.

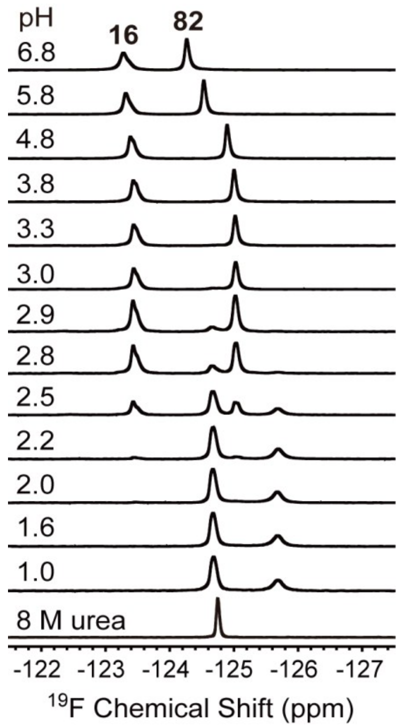

Figure S4. ^{19}F spectra of 5FW-labelled HdeA at different pH values and in 8 M urea.

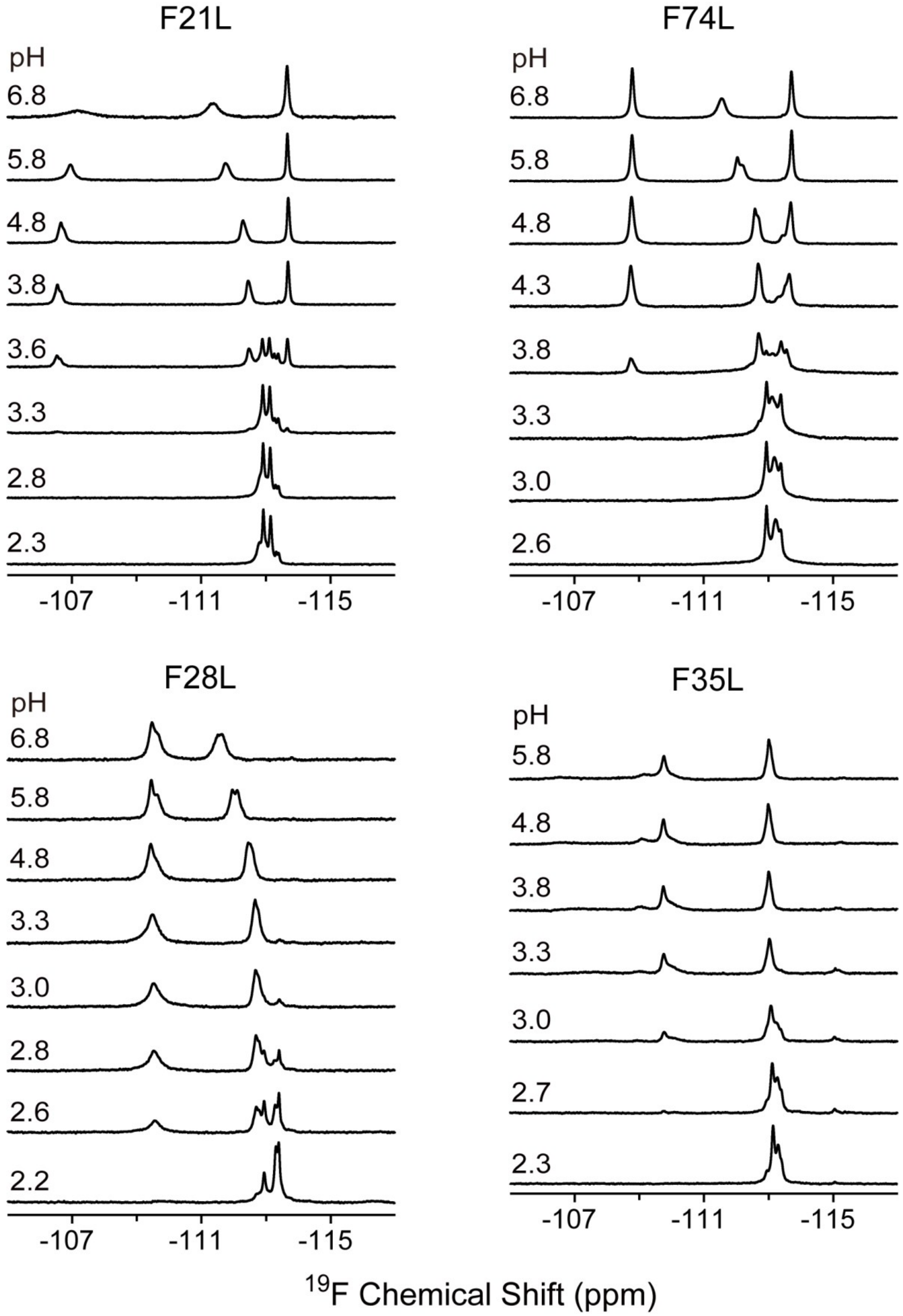

Figure S5. ^{19}F NMR spectra of 3FF-labelled HdeA variants (F21L, F74L, F28L and F35L) as a function of pH.

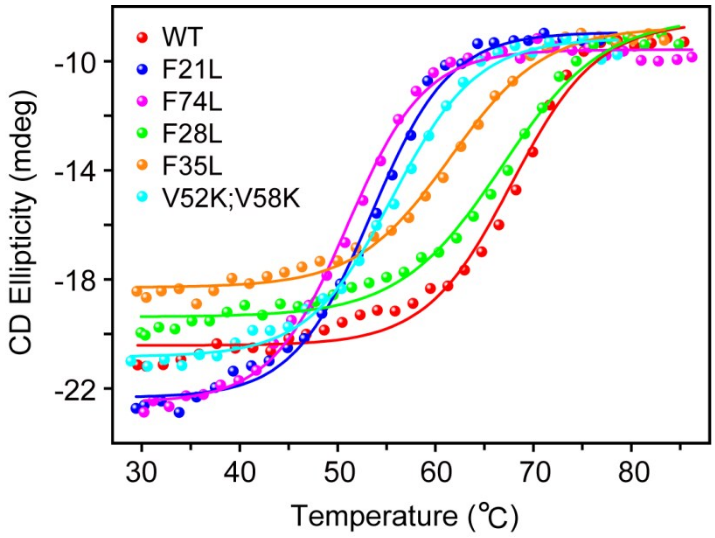

Figure S6. CD-monitored thermal unfolding of WT HdeA and several variants. The apparent melting temperature, T_m (see **Fig. 1d**), was obtained by fitting the thermal unfolding curves to a two-state model assuming linear baselines.

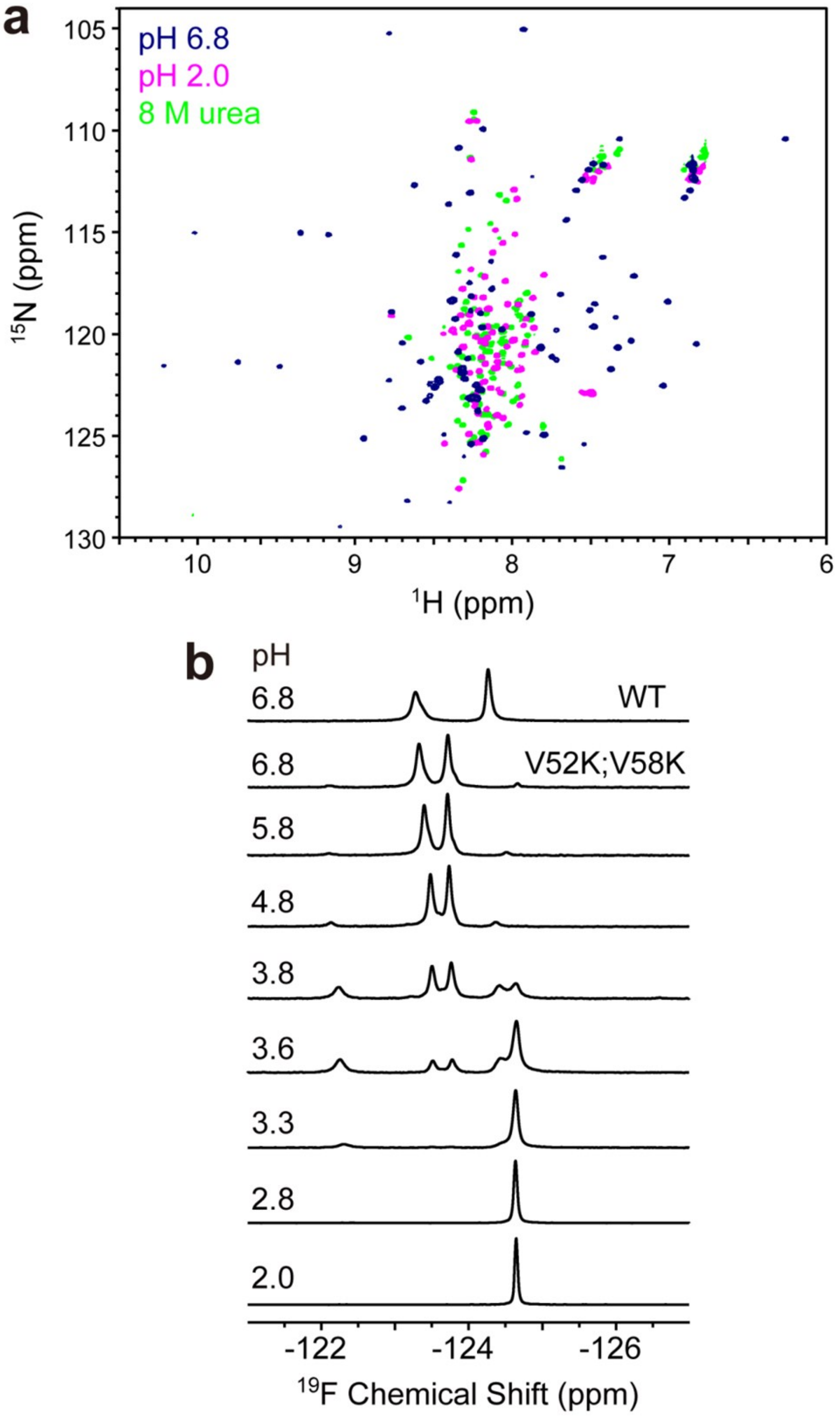

Figure S7. The V52K;V58K variant exhibits an acid-induced structural transformation similar to that of WT, but the transformation for the variant results in an unfolded structure that is distinctly different from the partially unfolded structure exhibited by WT at acid pH. Both ^{1}H-^{15}N HSQC (**a**) and ^{19}F spectra (**b**) demonstrate the transition from a folded to an unfolded structure as the pH is lowered. ^{19}F spectra also indicate that the variant exhibits an elevated transformation pH compared to WT. The ^{1}H-^{15}N HSQC spectrum of the variant at low pH is similar to the spectrum that in 8 M urea, indicating that the variant is completely disordered at pH 2.0, which is different from the residual structure observed for WT under these conditions (**Figure S1**).

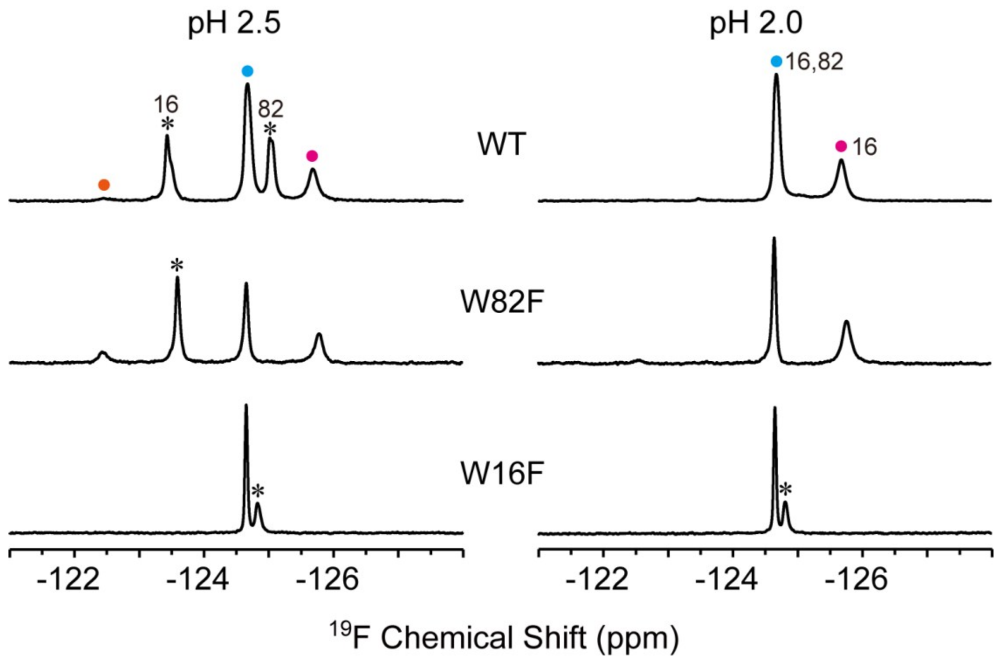

Figure S8. ^{19}F assignments of 5FW-labelled HdeA at low pH. The black asterisk indicates the original resonances at -123.6 ppm (W16) and -125.2 ppm (W82). Coloured circles indicate new resonances at -122.5 ppm (orange), -124.6 ppm (blue) and -125.8 ppm (magenta). The four resonances from W82F at pH 2.5 coincide with those of WT, except the W82 resonance is missing. The two peaks from W82F at pH 2.0 correspond to those from WT at pH 2.0. We conclude that W82 at pH 2.0 gives rise to a resonance at -124.6 ppm that overlaps one of the W16 resonances. In summary, for WT, the new narrower resonance (blue circle) comes from both W16 and W82, and the broader new resonances (orange and magenta circles) come from W16.

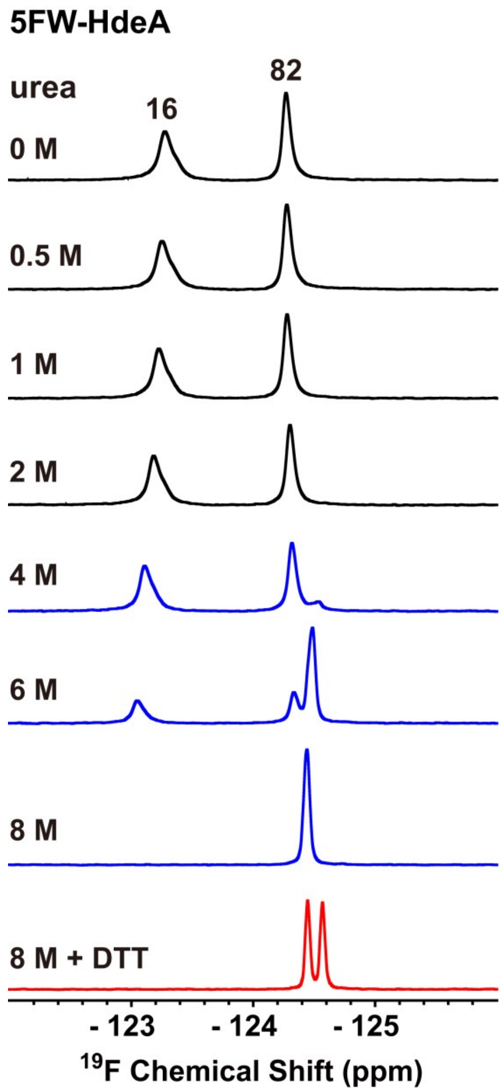

Figure S9. ^{19}F NMR spectra of 5FW labelled WT HdeA as a function of urea concentration. HdeA begins to unfold at 4 M urea. Unfolding by urea is different from unfolding by acid.

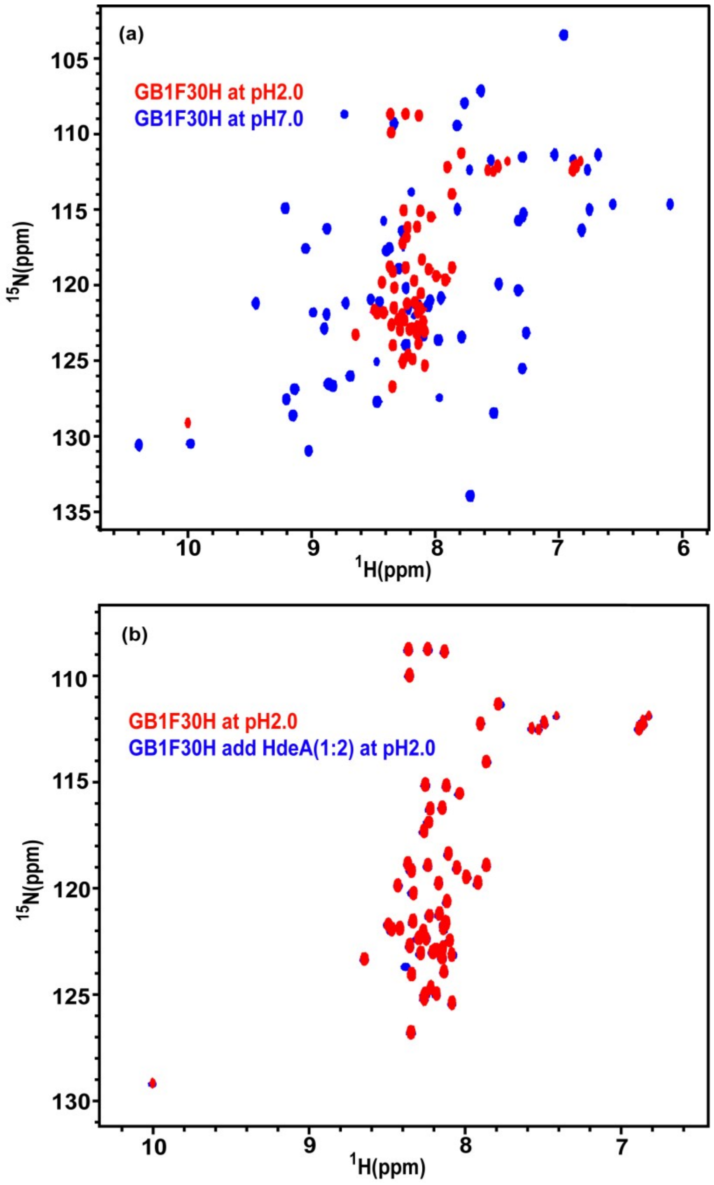

Figure S10. ^{1}H-^{15}N spectra of F30H GB1 at pH 7.0 and 2.0 (a), in the absence and presence of HdeA at molar GB1:HdeA ratio of 1:2 (b) at 37 °C.

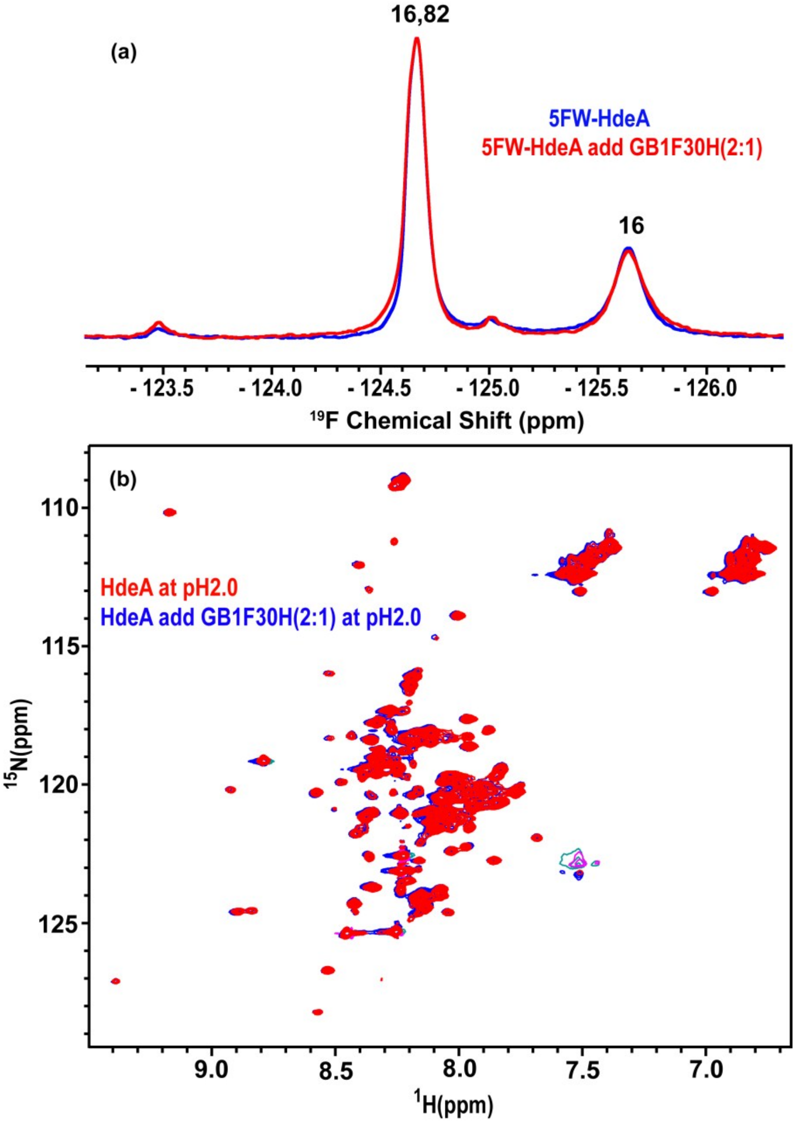

Figure S11. ^{19}F spectra (a) and ^{1}H-^{15}N HSQC spectra (b) of HdeA in the absence and presence of F30H GB1 at a molar GB1:HdeA ratio of 1:2 at 37 °C.

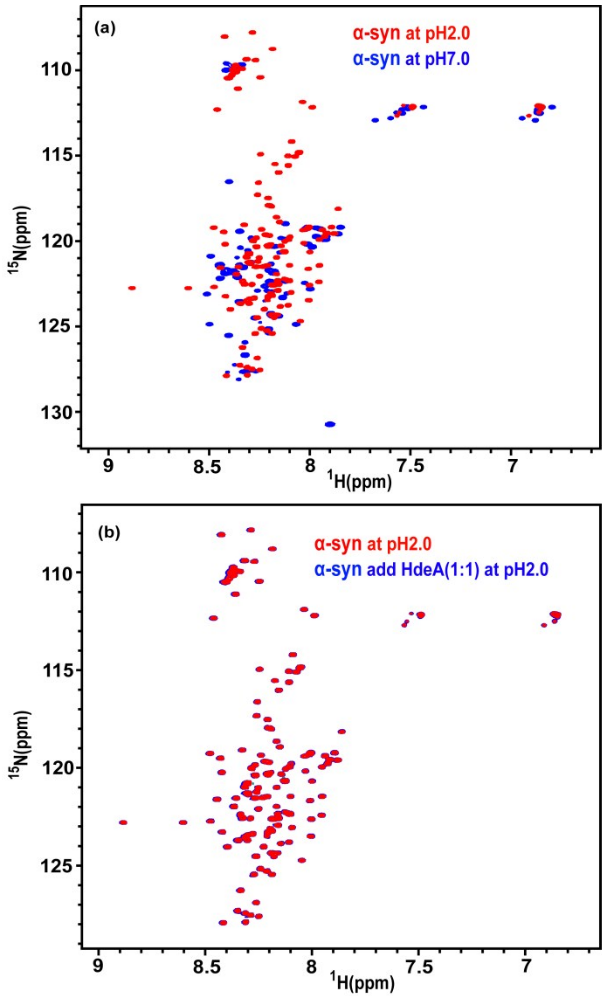

Figure S12. ^1H-^{15}N HSQC spectra of α-synuclein (αS) at pH 7.0 and 2.0 (a) in the absence and presence of HdeA at a molar αS:HdeA ratio of 1:1 (b) at 37 °C.

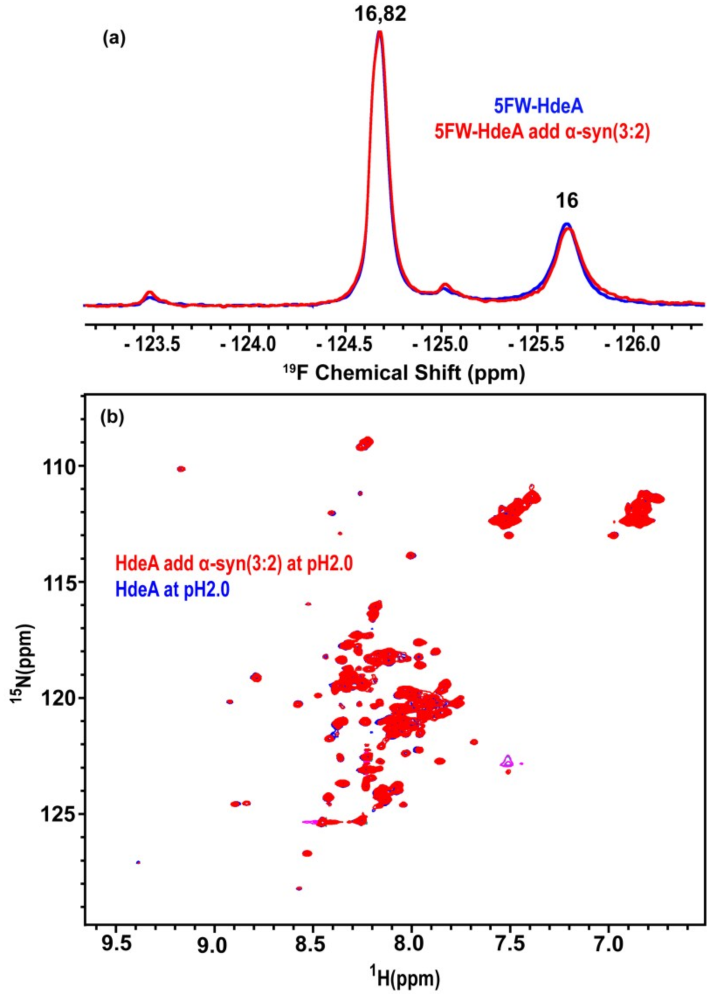

Figure S13. ^{19}F spectra(a) and ^{1}H-^{15}N spectra of HdeA in the absence and presence of αS at molar αS:HdeA ratio of 2:3 at 37 ^{o}C.

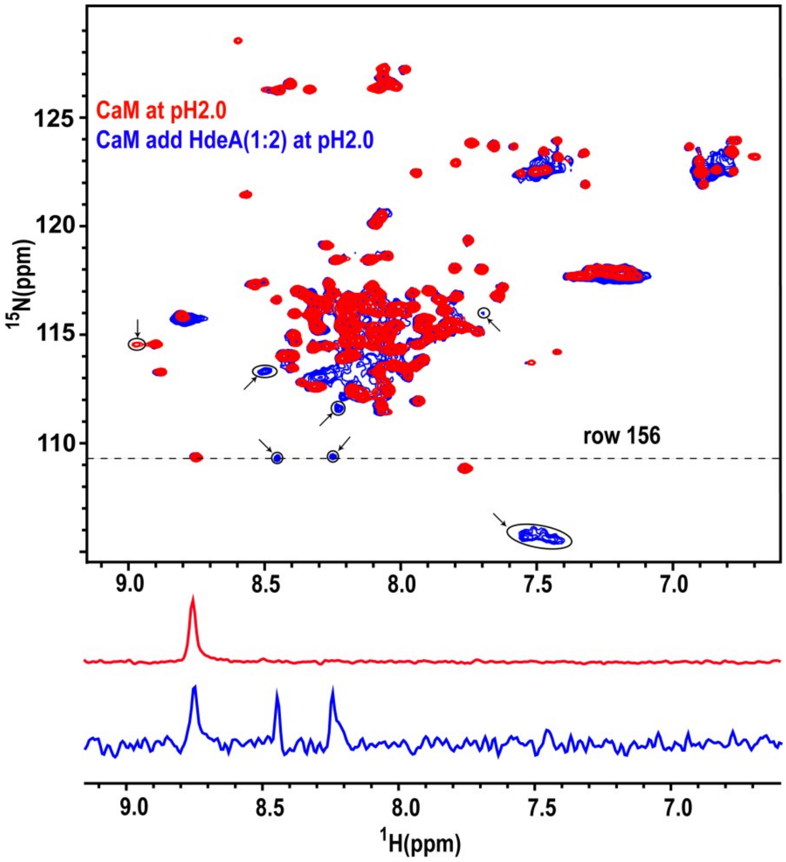

Figure S14. ^{1}H-^{15}N HSQC spectra of CaM in the absence and presence of HdeA at pH 2.0 and 37 °C. The bottom panel shows the indicated slices from the HSQC spectra.

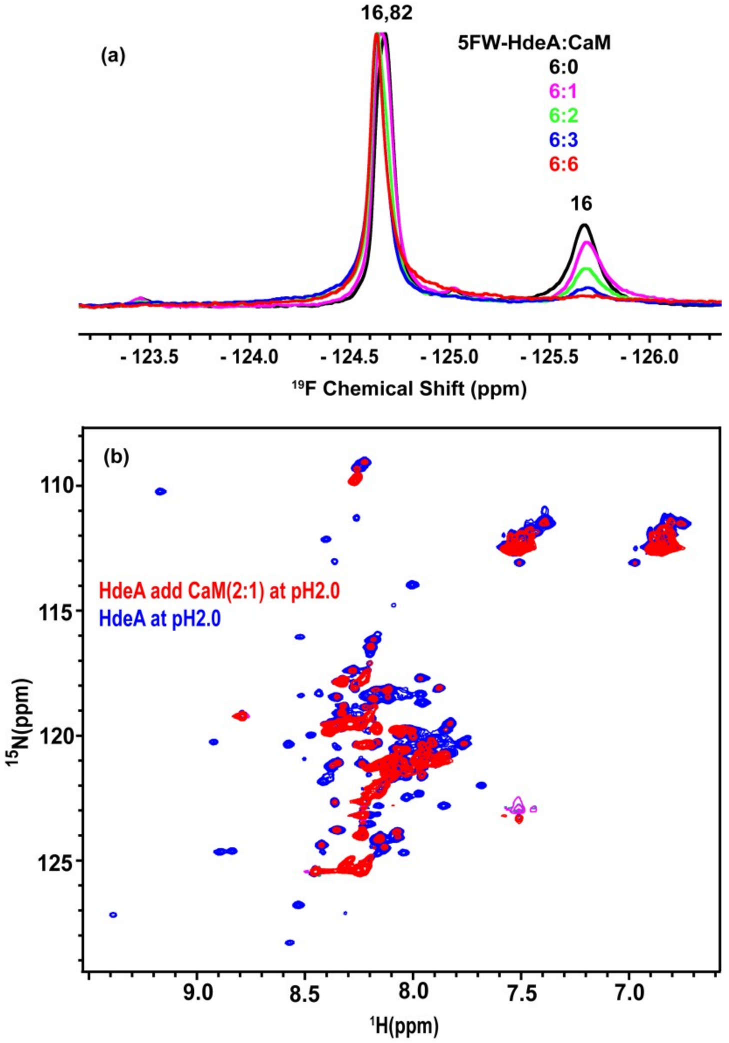

Figure S15. ^{19}F spectra (a) of HdeA upon adding CaM (molar 5FW-HdeA:CaM ratio from 6:0 to 6:6) and ^{1}H-^{15}N HSQC spectra of HdeA in the absence and presence of CaM at molar CaM:HdeA ratio of 1:2 and 37 °C.

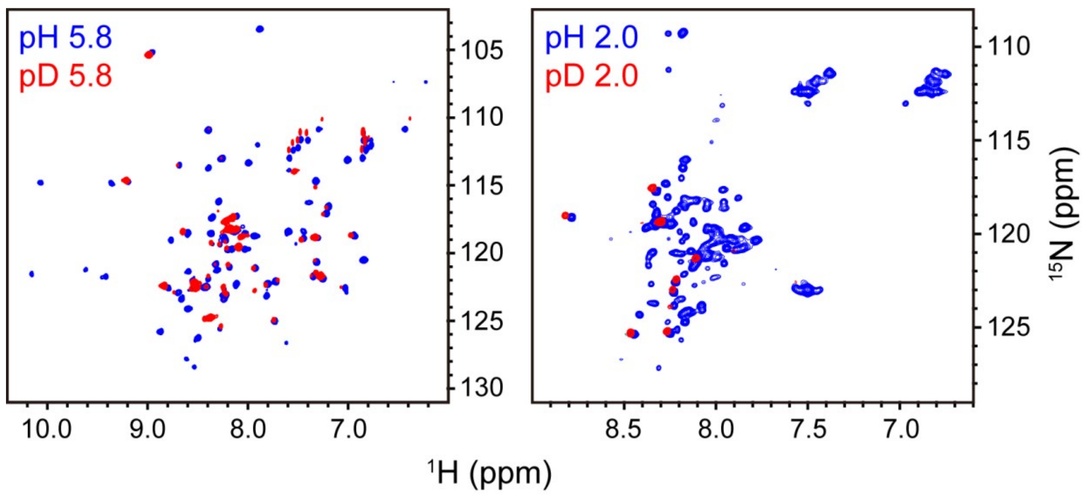

Figure S16. Hydrogen/deuterium exchange experiments reveal a partially folded structure of HdeA at acid pH. ^{1}H-^{15}N HSQC spectra of HdeA in 10% D_2O (blue) and 100% D_2O (red) at pH 5.8 and pH 2.0 after a few minutes and 2 h of exchange. At pH 5.8 many crosspeaks remain after 2 h of exchange, suggesting the presence of a well-folded structure at this pH. At pH 2.0, most crosspeaks are absent after 2 h, but several remain, indicating that some parts of the protein remain structured.

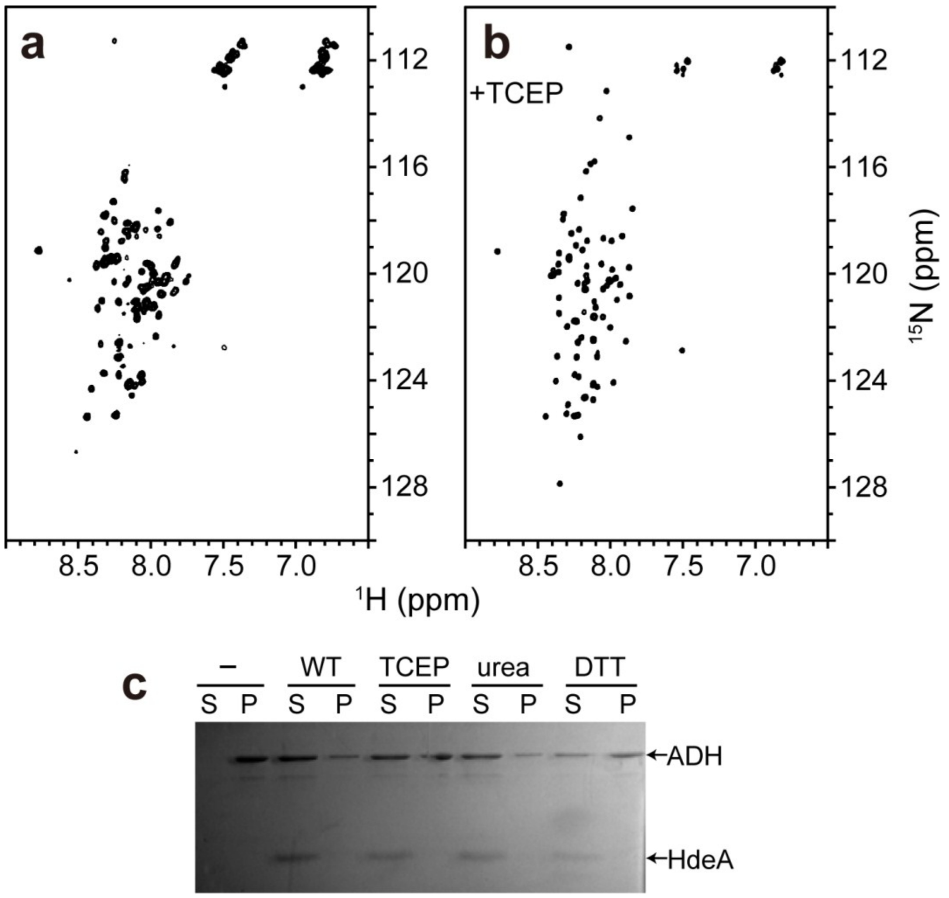

Figure S17. HdeA becomes disordered with greatly reduced chaperone activity when the intramolecular disulphide bond is reduced. **a, b,** ^{1}H-^{15}N HSQC spectra of WT and TCEP-reduced WT at pH 2.0. In contrast to the more disperse spectrum with broader cross peaks from the intact WT protein, reduced HdeA shows the sharp homogeneous spectrum characteristic of a disordered protein. **c,** Chaperone activity assays of WT. The substrate ADH was diluted to a final concentration of 10 μM in the assay buffer (pH 2.0) in the absence or presence of 30 μM untreated HdeA, HdeA treated with TCEP, HdeA treated with 8 M urea or treated with DTT and 8 M urea. The treated samples were diluted ten fold into assay buffer to ameliorate the effect of TCEP, urea and DTT. We used DTT to reduce the unfolded HdeA in 8 M urea at pH 7 for two reasons. First, DTT is not a reducing agent at acidic conditions. Second, the disulphide is buried in the structured dimer. The same result was observed with TCEP pretreatment at pH 2.0, suggesting greatly reduced chaperone activity upon reduction of the disulphide.
